# Effect of Nano-Clay and Surfactant on the Biodegradation of Poly(Lactic Acid) Films

**DOI:** 10.3390/polym12020311

**Published:** 2020-02-03

**Authors:** Pooja C. Mayekar, Edgar Castro-Aguirre, Rafael Auras, Susan Selke, Ramani Narayan

**Affiliations:** 1 School of Packaging, Michigan State University, East Lansing, MI 48824-1223, USA; mayekarp@msu.edu (P.C.M.); ragde.187@gmail.com (E.C.-A.); sselke@msu.edu (S.S.); 2 Department of Chemical Engineering and Material Science, East Lansing, MI 48824-1223, USA; narayan@msu.edu

**Keywords:** montmorillonite, surfactant, hydrolysis, bio-based, composting, biodegradable

## Abstract

This study examined the effect of nanoclays and surfactant on the hydrolytic degradation and biodegradation of poly(lactic acid) (PLA) and PLA nanocomposites. Organomodified montmorillonite (OMMT), unmodified montmorillonite (MMT) and an organomodifier (surfactant) for MMT (QAC) were extruded with PLA to produce PLA nanocomposites. The films were produced with the same initial molecular weight, thickness and crystallinity since these properties have a significant effect on the biodegradation process. The biodegradation experiments were carried out in an in-house built direct measurement respirometric system and were evaluated in inoculated vermiculite and vermiculite media for extended periods of time. Hydrolysis experiments were also conducted separately to decouple the abiotic/hydrolysis phase. The results showed no significant variation in the mineralization of PLA nanocomposites as compared to pristine PLA. The addition of nanoclays did not enhance the biodegradability of PLA when the initial parameters were strictly controlled. The hydrolysis test indicated that the nanoclays and surfactant did not aid in the degradation of PLA.

## 1. Introduction

Bio-based and biodegradable polymers have garnered great interest in the last decade as an alternative to the ever-growing demand for single-use petroleum based conventional polymers. Growing global concern regarding the environmental impacts and increasing awareness towards issues like plastic waste management and global climate change has led the consumers and in turn fueled governments and industries to adopt a greener or more sustainable approach [1]. Among the many waste management solutions, such as reducing, reusing, recycling and composting encouraged to reduce white pollution (i.e., single-use plastic contaminating the environment, reaching the oceans, nature and landfills), the development of biodegradable polymers has been a favorable one [2,3,4] since they can be recovered through recycling and composting. Poly(lactic acid)-PLA-a biobased and biodegradable plastic can provide an attractive solution to the waste disposal problem. Lactic acid (LA) is the precursor to polylactic acid (PLA) and can be obtained by the fermentation of renewable resources such as corn, potato and cassava starch [5,6].

PLA is a linear aliphatic polyester, which exhibits good stiffness, excellent barrier to flavor, good heat sealability, high clarity, ease of processing as well as grease and oil resistance comparable to conventional commodity plastics, such as polystyrene and poly(ethylene terephthalate) [7,8]. These attributes have promoted the use of PLA in various diverse commercial sectors, such as food, medical packaging, automotive, textile and agriculture [9,10,11]. However, other properties such as high permeability to gas and vapor, brittleness, low thermal stability and low melt strength have limited its widespread use [12]. Several approaches have been adopted to overcome these drawbacks of PLA [13,14,15]. An interesting approach to improve PLA’s properties is the reinforcement of PLA by addition of natural nanoclays, such as montmorillonite (MMT), resulting in PLA nanocomposites [16,17,18]. Due to low cost, easy availability and the significant enhancement in selective properties that MMT brings when added in low concentrations to PLA, these inorganic layered silicates or nanoclays have achieved wide acceptance [19,20,21].

MMT is an extensively studied filler belonging to the 2:1 phyllosilicate family of the smectite group with a chemical formula M_x_(Al_4−x_Mg_x_)Si_8_O_20_(OH)_4_·nH_2_O, where M is exchangeable cations such as Na^+^, K^+^, Li^+^ and Ca^2+^. The crystal lattice is made of two tetrahedral silica sheets fused to an octahedral sheet of aluminum by edge [22,23,24]. MMT is hydrophilic in nature due to the presence of hydrated inorganic cations. This nature hinders the dispersion of MMT in less hydrophilic organic polymers like PLA, tending to form agglomerates. Hence, surface modification of the hydrophilic MMT is necessary to convert it to an organophilic nanoclay, improving its affinity towards PLA. This is achieved by organophilization, a technique that is centered towards reducing the surface energy of nanoclays, such as MMT, thereby separating individual layers and improving its dispersion in the polymer matrix by better inclusion and percolation of the polymer chains [25,26]. Organomodified MMT (OMMT) is obtained by exchanging the inorganic cations of MMT with the surfactant organic cations including primary, secondary, tertiary and quaternary alkylammonium cations [27]. The surface energy of MMT is reduced due to the substitution of inorganic cations by organic ones, so the long alkyl tail increases the interlayer spacing thus improving the wettability of the polymer matrix [28].

Several studies have shown significant improvement in the properties of PLA because of the addition of nanoclays. Improvement in the barrier properties was observed and credited to the clays creating a more torturous path increasing the dwelling time for the permeants [21,29,30]. The presence of nanoclay was also reported to enhance the mechanical properties. Not only is it a stronger filler, the clay acts as a nucleating agent, thus increasing the crystallinity and in turn the tensile strength of the resulting PLA nanocomposite. Likewise, the elongation at break and the storage modulus (i.e., toughness) have been shown to improve due to the addition of nanoclays [31,32,33]. Lewitus et al., for example, reported a 30% increase in tensile modulus and 40% increase in elongation at break for PLA with the addition of OMMT as compared to pure PLA [34]. Ray et al., also recorded improvement of the mechanical properties and decreased gas permeability of PLA nanocomposites as well as an enhancement of the biodegradability of PLA [17,35,36].

Apart from shortcomings in some properties such as fragility and water barrier, another downside for PLA is that it degrades at a slower rate in comparison to other common natural organic wastes, such as food and yard waste, limiting its acceptance into industrial food and yard composting facilities [37]. The inclusion of OMMT was identified as one method to improve PLA’s properties when compared to their respective pristine polymers. In retrospect, the use of nanoclays has not only been reported to significantly improve the properties of biodegradable polymers but also to reduce the time it takes to biodegrade in the composting facilities [38,39,40]. This reduced biodegradation time frame makes it easier for biodegradable polymers such as PLA to be composted with other organic wastes, favoring its disposal through industrial composting facilities.

Nevertheless, it is still unclear how the nanoclays and organomodifiers (surfactant) affect the abiotic and biotic degradation mechanism of PLA since, for example, the addition of nanoclays is known to reduce the molecular weight of PLA modifying the start point of degradation [41]. So, comparison of PLA and PLA added with OMMT controlling for the initial molecular weight must be examined. Furthermore, the role of unmodified clay, organomodified clay and surfactant should be decoupled on the biodegradation steps, especially considering that the initial molecular weight is also affected due to the presence of these compounds.

Until now, addition of OMMT was claimed to induce faster biodegradation of PLA but no strict control was exercised over the main characteristics of the fabricated nanocomposite, such as the initial molecular weight, thickness and crystallinity, which significantly affect the biodegradation of PLA [35,42,43]. Thus, the overall goal of this study was to understand the role of MMT and surfactant on the biodegradation of PLA, when PLA and the PLA nanocomposites have the same starting conditions (i.e., molecular weight, thickness and crystallinity).

## 2. Materials and Methods

### 2.1. Materials

Poly(lactic acid), PLA 2003D (l-lactic acid content of 96%) with weight average molecular weight (*M*_w_) of 2.23 ± 0.04 × 10^5^ Da and number average molecular weight (*M*_n_) of 1.14 ± 0.07 × 10^5^ Da was procured from NatureWorks^®^ LLC (Minnetonka, MN, USA). Organomodified montmorillonite (OMMT), Nanomer^®^ I.34 TCN was obtained from Nanocor (Hoffman Estates, IL, USA) consisting of 80% montmorillonite (MMT) and 20% of surfactant (QAC). Tallow (b-hydroxyethyl) dimethyl ammonium chloride (QAC), the organo-modifier for OMMT was acquired from Haihang Industry Co. Ltd. (Jinan, China). The tallow is an alkyl group with approximately 65% C_18_H_37_, 30% C_16_H_33_ and 5% C_14_H_29_. Standard nanoclay polymer grade MMT was procured from Sigma-Aldrich (Highland, IL, USA). Tetrahydrofuran (THF) used as the mobile solvent phase to determine the molecular weight was obtained from Pharmco-Aaper (Brookfield, CT, USA). In the hydrolysis test, HPLC grade water was supplied by J.T. Baker (Center Valley, PA, USA).

### 2.2. Production of Nanocomposite Films

Masterbatches of PLA, PLA QAC, PLA MMT and PLA OMMT were produced in a co-rotating Century ZSK 30 twin-screw extruder (Century Extruders, Traverse City, MI, USA), pelletized and used for the production of the films. The PLA nanocomposite films were cast using a RandCastle RCP-0625 Multi-Layer Cast film extruder (Randcastle Extrusion Systems, Inc., Cedar Grove, NJ, USA). The processing of the masterbatches and films is further explained in the supporting information and the details of the processing conditions are provided in Table S1. The PLA, PLA QAC, PLA MMT and PLA OMMT films were processed in a controlled manner to have the same initial number average molecular weight (*M*_no_), crystallinity (%*X*_c_) and thickness. The PLA QAC film was produced at a slightly lower temperature range, since QAC acted as a plasticizer and also as a chain scission agent, reducing *M*_no_.

### 2.3. Characterization of PLA Nanocomposite Films

To measure the thickness and assess the surface morphology and dispersion of OMMT and MMT in PLA OMMT and PLA MMT films, scanning electron microscopy (SEM) and X-ray diffraction (XRD) was performed. SEM was used to evaluate the structure and determine the thicknesses of the films. Fractured samples were mounted on aluminum stubs and gold coated using an Emscope SC500 sputter coater (Emscope Laboratories, Ashford, UK) to improve the conductivity of the samples. The imaging and examination of the samples was carried out in a JEOL 6610V SEM (JEOL Ltd., Tokyo, Japan) at magnifications ranging from 5× to 50,000× at an accelerating voltage of 30 kV. XRD was used to investigate the samples on a Bruker AXS D8 Advance X-ray diffractometer (Bruker Co., Billerica, MA, USA) equipped with a Globel Mirror filtered Cu Kα radiation source setting of 40 kV and 100 mA. The film samples were analyzed and data was collected for 2θ range of 2° to 40° at a scan rate of 0.20°/min with 0.1° increment. The elemental analysis of all the films was performed using a Carbon/Hydrogen/Nitrogen analyzer (PerkinElmer 2400 Series II CHNS/O Elemental Analyzer, (PerkinElmer Inc., Shelton, CT, USA) as mentioned in Table S2 of the Supporting Information.

### 2.4. Biodegradation Test

The biodegradability of PLA, PLA QAC, PLA MMT and PLA OMMT films was evaluated using an in-house direct measurement respirometric (DMR) system by analysis of evolved CO_2_ under simulated composting conditions [44,45,46]. The aerobic biodegradation was performed in inoculated and non-inoculated vermiculite media at a controlled temperature of 58 ± 2 °C and relative humidity (RH) of 50% ± 5%. Vermiculite was used to overcome the priming effect which is observed in the compost media [47]. In addition to this, vermiculite acts as an inert inorganic matrix, thus reducing the background activity as opposed to compost media and improving the test dependency. The use of activated vermiculite does not alter the biodegradation rate of the polymers tested. Thus, activated vermiculite was used in place of mature compost to simulate composting conditions [48]. The evolved CO_2_ concentration was measured using a non-dispersive infrared gas analyzer (NDIR) Li-COR^®^ LI-820 (Licor Inc., Lincoln, NE, USA). Manure compost was obtained from Michigan State University (MSU) Composting Facility (East Lansing, MI, USA). Prior to use, the compost was sieved through a 10 mm mesh to remove any large particles that might be present and conditioned at 58 °C for 3 days. Vermiculite, a hydrous phyllosilicate mineral of premium grade, was purchased from Sun Gro Horticulture Distribution Inc. (Bellevue, WA, USA). Deionized water was added to dry compost and stirred vigorously and the mix was allowed to settle for 30 min. The solids were then separated through a 1 mm mesh to obtain the compost extract. This compost extract was then amalgamated with a mineral solution in 1:1 ratio resulting in the inoculum solution. Detailed information regarding the mineral solution preparation can be found elsewhere [49]. The vermiculite was mixed with the inoculum solution in the ratio of 1:4 parts and with distilled water in the same ratio to obtain inoculated and non-inoculated vermiculite media, respectively. The physicochemical parameters such as dry solids, pH, volatile solids, C/N ratio and nutrients were determined by the Soil and Plant Laboratory at MSU (East Lansing, MI, USA). A complete explanation of the methods used to determine the physicochemical parameters of the compost are described elsewhere [49]. The bioreactors (~2 L) were packed with 400 g of the media (either inoculated vermiculite or vermiculite) mixed rigorously with 8 g of film samples (1 cm × 1 cm pieces) to be tested, leaving enough headspace (about ¼ of total volume). Triplicate bioreactors of each sample variant were placed in an environmental chamber, simulating composting conditions, where the evolved CO_2_ was measured and recorded. An additional bioreactor was run in each case to evaluate the molecular weight reduction. The CO_2_ evolution of blank samples, that is, bioreactors filled only with either inoculated vermiculite or vermiculite, was also measured. Similarly, cellulose was used as a positive control reference because of its high biodegradability.

CO_2_ free (<30 ppm) water saturated air (50% ± 5% RH) was supplied to each bioreactor during the test. The CO_2_ evolved from the bioreactor was measured by the NDIR sensor at uniform intervals. The measuring system was purged throughout by CO_2_ free air to remove any residual traces after every measurement and before measuring the next bioreactor. Mineralization, which is defined as total amount of carbon converted to CO_2_ molecules, was calculated using Equation (1).

(1) $$Mineralization\;\%=\;\frac{(CO_{2})t-\;(CO_{2})b}{M_{t}\;\times \;C_{t}\;\times \;\frac{44}{12}}\;\times \;100$$

The numerator is the difference between the average cumulative CO_2_ mass evolved from the sample (*CO*_2_)*t* and the average CO_2_ evolved from the three blank bioreactors (*CO*_2_)*b*. The theoretical amount of CO_2_ ideally able to be produced by the sample is the denominator, wherein *M_t_* is the total mass of the sample, *C_t_* is the amount of carbon present in the sample as derived from CHN analysis, 44 is the molecular mass of CO_2_ and 12 is the atomic mass of carbon.

### 2.5. Molecular Weight Determination

The samples were collected every 5 days to determine the number average molecular weight (*M*_n_), weight average molecular weight (*M*_w_) and dispersity (Đ) of PLA and PLA nanocomposite films. Samples weighing approximately 0.01 g were dissolved in THF at a concentration of 2 mg/L. The samples were run using a size exclusion chromatography (SEC) unit (Waters Corp, Milford, MA, USA) equipped with a Waters^®^ 1515 isocratic pump, Waters^®^ 717 autosampler, a series of Waters^®^ Styragel columns HR4, HR3, HR2 (300 mm × 7.8 mm (I.D)) and a Waters^®^ 2414 refractive index detector. The columns were maintained at a temperature of 35 °C and THF was run at a flow rate of 1 mL/min. The molecular weight distribution (MWD) was analyzed using Waters^®^ Breeze software. A third order polynomial calibration curve was derived using polystyrene (PS) standards with *M*_w_ in the range of 500–2.48 × 10^6^ Da. The Mark -Houwink constants of *K* = 0.000164 dL/g and α = 0.704 were used to obtain absolute *M*_n_ and *M*_w_ of PLA. Deconvolution of the MWD peaks was carried out to find the underlying populations of molecular chains making up the peaks. This was performed using Fityk 1.3.0, a curve fitting and data analysis program, developed under the terms of the GNU General Public License for nonlinear fitting of a LogNormal function to experimental MWD. The area fraction methodology was used wherein the peak with maximum area was selected as a representative of the majority of chain length as previously developed by the authors [50,51].

### 2.6. Hydrolysis Test

A hydrolysis test was run to understand the hydrolytic degradation of the PLA and PLA nanocomposite films and was adapted from ASTM D4754-18 [52]. The hydrolysis cell consisted of a glass vial with a cap, stainless steel wire and glass beads. Ten discs of PLA, PLA QAC, PLA MMT or PLA OMMT cut into 2 cm diameter discs were threaded onto a stainless-steel wire and separated using glass beads. A schematic representation depicting the cell is provided in Section S5, Figure S5 of the Supporting Information. The discs were then stored in water (HPLC grade) (J.T. Baker, Center Valley, PA, USA) media, previously conditioned at 60 °C and the hydrolysis test was performed. The ratio of the surface area of the disc to the fluid volume was 1.81 cm^2^/mL. Samples were retrieved in triplicates and dried before assessing *M*_n_ and *M*_w_ using SEC to find the hydrolytic degradation rate constant. Also, the degree of crystallinity (%*X_c_*) was determined using the first heat scan, using differential scanning calorimetry (DSC) as explained below.

### 2.7. Thermal Properties

Thermal gravimetric analysis was performed to determine the heat stability of the samples, determined by the weight loss of the samples as a function of temperature. This was investigated on a Q-50 thermogravimetric analyzer (TA Instruments, New Castle, DE, USA). The samples weighed between 5 and 10 mg and were heated to 600 °C, at a ramp rate of 10 °C/min under high purity nitrogen atmosphere (70 mL/min) to avoid thermoxidative degradation. A DSC Q100 (TA Instruments, New Castle, DE, USA) was used to determine the glass transition temperature (*T*_g_), melting temperature (*T*_m_), crystallization temperature (*T*_c_) and % *X_c_*. The variation in the crystallinity of the samples during hydrolytic degradation was determined from the first heating cycle. Samples weighing between 5 and 10 mg were sealed in aluminum pans cooled to −5 °C and then heated to 180 °C at a ramp rate of 10 °C/min. The nitrogen purge flow was maintained at a constant rate of 70 mL/min. The thermograph was analyzed using the software Thermal Universal Analysis 2000, V4.5 (TA Instruments). The % *X*_c_ was calculated using Equation (2).

(2) $$\%\chi_{c}=\;\frac{\Delta H_{m}-\Delta H_{c}}{\Delta H{{}^{\circ}}_{m}\left(1-\frac{{\%\;\mathrm{wt}}_{\mathrm{filler}}}{100}\right)}\;x\;100$$

where $\Delta H_{m}$ is the heat of fusion, $\Delta H_{c}$ is the enthalpy of cold crystallization and $\Delta H{{}^{\circ}}_{m}$ is the enthalpy of fusion for 100% pure crystalline PLA (93 J/g) and ${\mathrm{wt}}_{\mathrm{filler}}$ is the weight fraction of OMMT, MMT and surfactant in the PLA OMMT, PLA MMT and PLA QAC films, respectively. The results for thermal properties are described in Figures S1, S2 and Table S3 in Section S3 of the Supporting Information.

### 2.8. Statistical Analysis

All the statistical analyses were conducted using MINITAB^TM^ 18 software (Minitab Inc., State College Park, PA, USA). One-way ANOVA and Tukey’s test were used to evaluate statistical significance at *p* < 0.05. All the values are reported as mean ± standard deviation.

## 3. Results

To understand the influence of nanoclays and surfactant on the hydrolytic degradation and biodegradation of PLA and PLA nanocomposites, PLA, PLA QAC, PLA MMT and PLA OMMT films were produced. The films were characterized for their initial molecular weight, thickness and crystallinity. To determine the rate at which the biodegradation takes place, the films were introduced in non-inoculated vermiculite and inoculated vermiculite media at 58 ± 2 °C and the reduction in *M_n_* was analyzed. The films were also exposed to water media at 58 ± 2 °C to determine how the nanoclays and surfactant affect the hydrolytic degradation. The rate of hydrolytic degradation and biodegradation was studied by collecting samples from the media at specific time intervals. A deconvolution technique was implemented to find the main population of the molecular chains representing the average *M*_n_ and to understand the depolymerization kinetics. In addition, the evolution of crystallinity of films exposed to water was also monitored.

### 3.1. Characterization of All Films

Table 1 shows the initial value of *M*_no_, *M*_wo_, thickness, as measured by SEM, and crystallinity for PLA, PLA QAC, PLA MMT and PLA OMMT films. The films were initially fully characterized since one of the main objectives was to produce films with no more than 10% variation of *M*_no_, %*X*_c_ and thickness to understand the effect of nanoclays and surfactant on the hydrolytic degradation and biodegradation process. We deliberately set variation at 10% because it was very difficult to achieve all the parameters with lower variation. No significant difference was observed for PLA, PLA MMT and PLA OMMT films in terms of *M*_no_; however, *M*_no_ of PLA QAC films was slightly lower than the other films. This can be attributed to the presence of water. Despite drying the PLA pellets before processing, there is some amount of moisture ingress during laboratory processing. During the extrusion process, water attacks the ester group (–COO) of PLA leading to chain scission and PLA degradation [53]. Each ester bond in the polymer has an equal chance of undergoing random chain scission whereas only the ester groups at the end of the polymer chains can undergo end scission. Random chain scission can occur at any point of the polymer chain and hence one scission can drastically reduce PLA’s molecular weight [54]. End scission reduces the polymer chain length by one unit, which is not sufficient to bring a remarkable drop in the molecular weight. The presence of QAC triggers a certain degree of hydrolysis of the PLA matrix due to the random chain scission happening at high temperatures during the extrusion process. A suggested reaction mechanism is shown in Section S2 of SI. This results in the global reduction of molecular weight of PLA. No variability was observed in the thicknesses of the films. Additional information regarding the use of SEM for the measurement of thickness and the SEM images for all the films is provided in Section S3, Table S4 and Figure S3 of the SI. The %*X*_c_ crystallinity of all the films was low and no significant difference was seen, ensuring that the films were initially amorphous as also confirmed by XRD analysis and shown in the Supporting Information Section S4, Figure S4 of the SI.

### 3.2. Biodegradation: CO_2_ Evolution and Mineralization in Non-Inoculated and Inoculated Vermiculite

**Figure 1:**
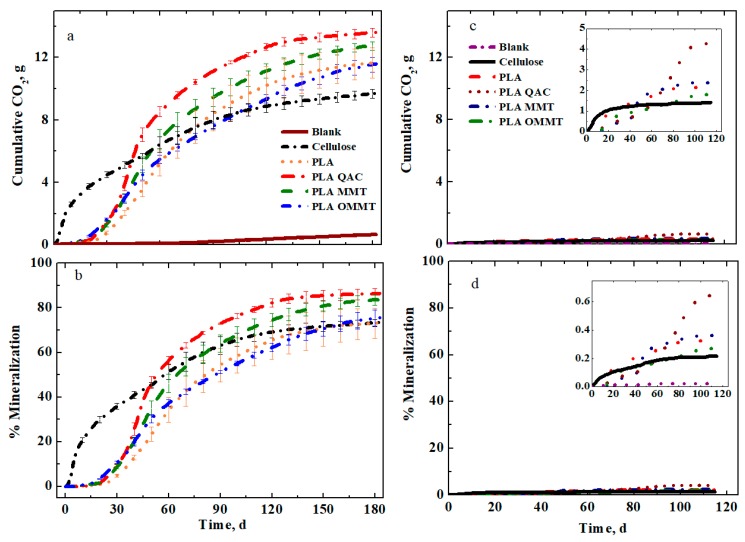
CO_2_ evolution (**a**) and % Mineralization (**b**) of PLA, PLA QAC, PLA MMT and PLA OMMT films in inoculated vermiculite and CO_2_ evolution (**c**) and % Mineralization (**d**) for non-inoculated vermiculite.

The biodegradability of PLA, PLA QAC, PLA MMT and PLA OMMT films was monitored by CO_2_ evolution using the in-house DMR and conducted for 180 days and 120 days for inoculated and non-inoculated vermiculite, respectively. Since the rate at which biodegradation proceeds in the inoculated and non-inoculated vermiculite is slow as compared to compost media, the test was conducted for an extended period of time compared to compost testing (between 90 and 120 days). The films were tested in the inoculated and non-inoculated vermiculite to avoid the priming effect, which is commonly observed in compost media [47,48]. There is also no indigenous carbon available in vermiculite as it is an inert solid media compared to compost media. Additionally, vermiculite produces very low variation among the replicates. The CO_2_ evolution and mineralization for all the films in the inoculated vermiculite is depicted in Figure 1a,c. The blank bioreactors contain only the solid media that is, inoculated and non-inoculated vermiculite and no polymer films. The CO_2_ evolution in the blank bioreactors is negligible due to the absence of carbon material. Along with the films, cellulose powder was also introduced in the DMR as a positive reference material since it is well-known that it is easily biodegradable.

Three dominant phases of biodegradation that is, lag phase, biodegradation phase and plateau phase, can be observed. Cellulose reached a maximum mineralization of 73.3% and evolved 9 g of CO_2_ by the end of the test (Figure 1b). The hydrophilic nature of cellulose combined with the action of extracellular enzymes available in the natural environment, reduces it to a size at which it is easily transported through the cell wall of micro-organisms for easy assimilation by metabolic pathways [49]. Due to this, the lag phase for cellulose powder is insignificant and the biodegradation phase starts almost from the beginning of the test. The lag phase is defined as the time in days, from the start of the test until the time of activation of the degrading micro-organisms is achieved. The lag phase is determined from the graph when a steep increase in evolution of CO_2_ is observed. A lag phase of 25 days was estimated for PLA due to the initial hydrolytic degradation. The hydrolytic degradation proceeds by the cleavage of ester bonds by water. The cleavage of molecular chains causes PLA to break into shorter fragments generating smaller chains. As a result, lactic acid oligomers are generated, which can then be easily consumed by the micro-organisms producing CO_2_ and water [55,56]. The hydrolysis phase in PLA is essential for the breakdown of the longer and high *M*_n_ PLA chains into lower *M*_n_ chains for easy assimilation by micro-organisms. Castro-Aguirre et al. concluded that PLA chains with *M*_n_ < 10 kDa are assimilated by micro-organisms after the initial hydrolysis step [49]. On the other hand, PLA QAC and PLA MMT showed a lag phase of 20 days, while PLA OMMT showed 15 days. The reduction in lag phase as compared to PLA in the case of PLA QAC could be attributed to the presence of hydroxyl groups in the clay organic modifiers (QAC) and also lower starting initial molecular weight (*M*_no_ ≤ 100 kDa). For PLA OMMT, the reduced lag phase could be explained by the relatively higher hydrophilicity of the nanoclay induced by the presence of QAC (~27 wt %) The values reported for the lag phase are in accordance with the values published earlier in the literature [57]. PLA QAC produced 13.6 ± 0.3 g of CO_2_, whereas PLA OMMT, PLA MMT and PLA produced 11.6 ± 0.5 g, 12.8 ± 0.3 g and 11.7 ± 1 g of CO_2_, respectively by the end of the test (180 days). Maximum mineralization of 86.4% was observed for PLA QAC, followed by PLA MMT at 83.6% and then PLA OMMT and PLA with 75.5% and 73.0%, respectively. As seen in Figure 1b, the mineralization curves for all the films were similar until day 30 and no major visible difference was seen in the lag phase and initial biodegradation phase, mostly driven by hydrolysis. But as the test continued, starting at day 50 the PLA QAC film showed a rapid increase in biodegradation compared to PLA OMMT and PLA films. This was due to the low initial molecular weight, which proved as an added advantage for PLA QAC film to reach early the needed *M_n_* for engaging the biodegradation stage. But, as the biodegradation phase proceeded, all the films reached the plateau phase around the same time of approximately 150 days. By the end of the test, no significant difference was seen with respect to mineralization among the PLA, PLA QAC, PLA MMT and PLA OMMT films (i.e., there was no improvement in the biodegradability of PLA due to the addition of nanoclays or the setup of this experiment did not have the power to detect any difference). By producing and testing PLA QAC films along with the PLA MMT and PLA OMMT films, the effect of QAC on the biodegradation rate of PLA was accounted for. Some researchers claimed that the addition of nanoclays enhanced the degradation rate of PLA nanocomposites as compared to pristine PLA [58,59,60]. Our finding is in disagreement with the results published in literature so far. This is due to the fact that our experiments accounted for the same initial parameters, which was not considered in the previous studies. Also, it will be of interest to evaluate the results of biodegradation for PLA, PLA QAC, PLA MMT and PLA OMMT films with equal weight of PLA, which were not conducted in this work.

Figure 1c,d show the results when the films were tested with the non-inoculated vermiculite. Since no micro-organisms were present in the media, no significant difference was seen in the CO_2_ evolution during the abiotic degradation. Also, no significant difference was observed in the mineralization values. The minute degradation seen (4.3% mineralization for PLA QAC), can be ascribed to the abiotic hydrolytic degradation process and minor contamination through the humidification of the air stream in the equipment since it is very challenging to maintain a sterile environment for 120 days. Details of this equipment can be found elsewhere [49].

### 3.3. MWD and M_n_ Reduction of PLA, PLA QAC, PLA MMT and PLA OMMT in Different Medias

**Figure 2:**
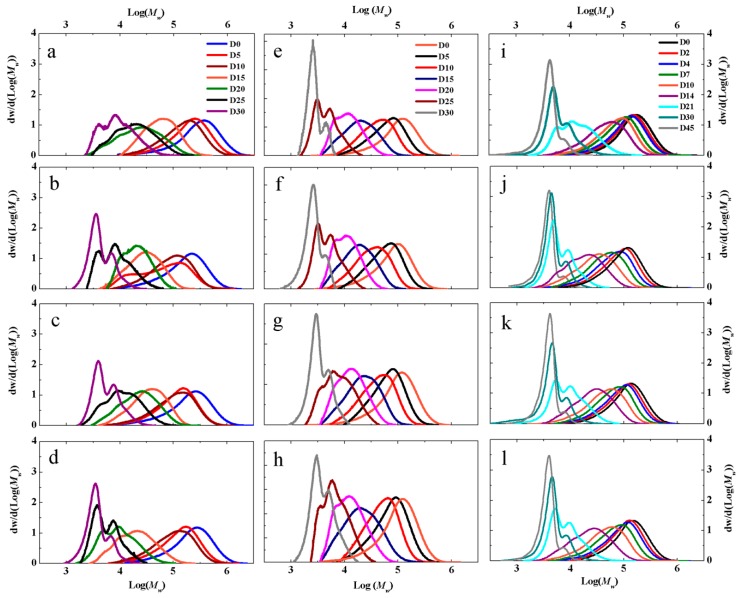
Change in molecular weight of (**a**) PLA, (**b**) PLA QAC, (**c**) PLA MMT and (**d**) PLA OMMT films in inoculated vermiculite media, (**e**) PLA, (**f**) PLA QAC, (**g**) PLA MMT and (**h**) PLA OMMT films in non-inoculated vermiculite media and (**i**) PLA, (**j**) PLA QAC, (**k**) PLA MMT and (**l**) PLA OMMT in water.

To better understand the effects of nanoclays and surfactant on biodegradation stages, film samples were retrieved from the bioreactors (inoculated and non-inoculated vermiculite media) and hydrolysis test vials (water media) at specified time intervals to track the changes in molecular weight. Figure 2 illustrates the MWDs for PLA, PLA QAC, PLA MMT and PLA OMMT exposed to inoculated vermiculite, non-inoculated vermiculite and water. The majority of the samples were collected during the first 30 and 45 days of the biodegradation experiments, mostly reflecting the *M*_n_ changes during the initial hydrolysis process.

Deconvolution of the MWD peaks was conducted to better understand the underlying reaction kinetics and mechanisms. The deconvolution of peaks was performed using the LogNormal function when skewness in the MWD and when more than one peak was detected to determine the rate constants. As seen in Figure 2, as the biodegradation in inoculated and non-inoculated vermiculite and hydrolysis in water proceeds, the MWD profiles in general shift to the left, depicting a decrease in *M*_n_ while the broadening of the peaks portrays an increase in the dispersity (Đ) originating due to the random chain scission and end chain reactions producing lactic acid oligomers and monomers [56,61]. When more than one peak that is, multimodal distribution (bimodal, trimodal or n-modal) was observed in the MWD, it can be attributed to the combination of two or more underlying chain populations and different underlying biodegradation process. The shifting of the main peak is observed during the abiotic phase that is, during the hydrolytic degradation and is mainly accredited to the random chain scission happening in the bulk of the polymer and not just on the surface [62,63]. If the hydrolysis proceeded via surface erosion, the main peak would stay at the same starting position with reduced peak area. The fact that the main peak moves away from its original position towards the low molecular weight side indicates a major bulk erosion phenomena [64]. As the hydrolysis progresses in all medias, broadening of the MWD peak is seen overall. The change from a single peak to multiple peaks at the later stage can be attributed to the development of crystal residues, resulting due to the repositioning of the newly formed short chains. The main peak is replaced by a small shoulder at the original molecular weight position and a comparatively large shoulder at low molecular weight. The peak corresponding to high molecular weight vanishes, leaving behind a sharp low molecular weight peak (for example Figure 2b,e,k). The amorphous fraction is subjected to the main hydrolysis while the crystalline residue seems to remain stable. This is corroborated by several studies, which indicate preferential degradation of amorphous regions during hydrolysis [65,66,67]. However, the full biodegradation mechanism of PLA (i.e., initial hydrolysis through random and end chain scission and following by biodegradation) is still not completely understood.

Multimodal peaks start to appear for PLA, PLA QAC, PLA MMT and PLA OMMT films starting day 20 irrespective of the media, indicating the dominance of different low molecular weight species. More defined and sharp peaks are observed by the end of the test around day 30 indicating that the degradation of amorphous regions and lower *M*_n_ takes place, predominantly [39,68,69]. Much sharper and more pronounced peaks are seen for PLA QAC, PLA MMT and PLA OMMT as compared to PLA in inoculated vermiculite media. In the case of PLA MMT and PLA OMMT films, this could be due to the nanoclays acting as a nucleating agent of the shorter PLA oligomers. Due to its large surface area, MMT may be possibly enhancing the degradation of the amorphous regions and promoting the formation of shorter chains, which can then crystallize [42]. Between days 25 and 30, it can be noticed that there is no remarkable shift of the peak to the left; rather the peaks become taller and sharper, implying degradation of the amorphous regions and consumption of low molecular weight chains by the micro-organisms. The *M*_n_ values for PLA, PLA QAC, PLA MMT and PLA OMMT were approximately 9, 3.4, 8 and 2.9 kDa respectively at day 30.

For non-inoculated vermiculite, the same behavior is observed among all the films, though a clear difference can be seen in the peak heights as compared to inoculated vermiculite. The peak at day 30 is predominant and became much higher and sharper for non-inoculated vermiculite as compared to the peak at day 30 for inoculated vermiculite for PLA, PLA QAC, PLA MMT and PLA OMMT films (Figure 2e–h). This can be due to the fact that no micro-organisms are present in the non-inoculated vermiculite, to be able to consume low *M*_n_ PLA chains and the degradation is purely based on hydrolysis that is, abiotic degradation. The increase of the sharp peaks is currently mostly attributed to the formation of highly ordered crystalline regions, resulting from the cleavage of ester bonds and random chain scission. The *M*_n_ values for PLA, PLA QAC, PLA MMT and PLA OMMT were approximately 2.6, 5.2, 2.8 and 2.9 kDa respectively at day 30. This finding opens the research query about the relative assisted early biodegradation of high *M*_n_ PLA during the hydrolysis stage due to the presence of micro-organisms.

In the case of the water media, the transition of peaks from a narrow MWD to a broad MWD was smooth as compared to inoculated vermiculite probably due to the absence of solid media. The peak stays at the same place after day 21 and starts to become sharper and narrower. Even in the presence of water exclusively, no statistical difference was found in the degradation rate of all the films. This can be due to the fact that when all the films have the same starting parameters and are exposed to the same conditions, the presence of surfactant and nanoclay may not enhance the degradation rate. Overall across all media, no significant difference was observed due to the addition of nanoclays, irrespective of the media.

**Figure 3:**
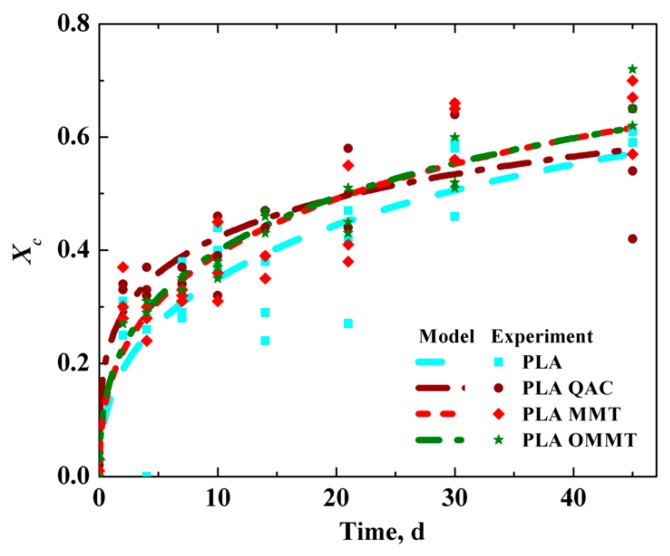
Crystalline fraction of PLA, PLA QAC, PLA MMT and PLA OMMT during hydrolytic degradation at 60 °C. Fitted lines were estimated from the Avrami equation, *X*_t_ = 1 − exp[−(*k*_c_*t*)*n*_c_], where *X*_t_ is the time-dependent fraction of crystallinity, *k*_c_ is the Avrami constant, *t* is the hydrolysis time and *n*_c_ is the Avrami exponent.

For the water media, the samples were tested for the change in crystallinity over the period of hydrolysis (Figure S6). The *X*_c_ increased from 3.7% to 60.1% for PLA and from 1.0% to 54.0% for PLA QAC. Similarly, a considerable increase in crystallinity was observed for PLA OMMT and PLA MMT film. The *X*_c_ for PLA OMMT reached 66.3%–71.4% and for PLA MMT it was 64.7%–71.5% at day 45 as seen in Figure 3. The *X*_c_ of the films was estimated using the Avrami equation. The hydrolytic degradation of all films in water media shows some fraction of crystalline region present by day 45, irrespective of starting out completely amorphous at day 0.

**Figure 4:**
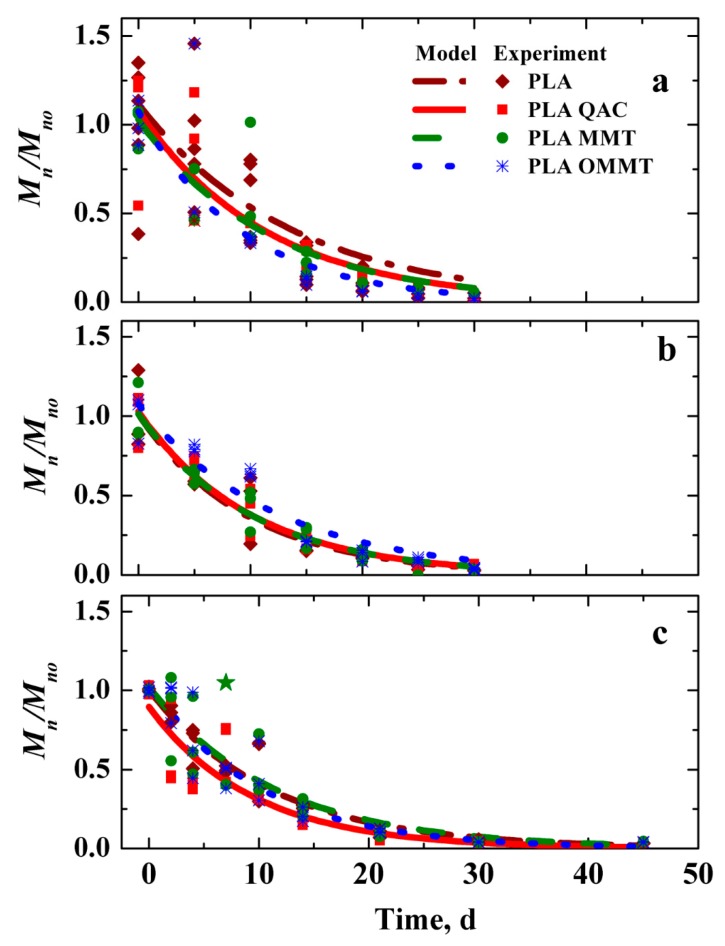
Reduction in molecular weight as a function of time for PLA, PLA QAC, PLA MMT and PLA OMMT films in (**a**) inoculated vermiculite (**b**) non-inoculated vermiculite and (**c**) water media. Values indicated as ★ were considered as outliers and hence not used for fitting. Fitting of the first order equation *M*_n_/*M*_no_ = e^(−*kt*)^, where *M*_no_ is the initial *M*_n_, *k* is the rate constant and *t* is the time.

Figure 4 shows the reduction in *M*_n_/*M*_no_ for PLA, PLA QAC, PLA MMT and PLA OMMT films as a function of time. The samples could be collected from the inoculated and non-inoculated vermiculite only until day 30 but for hydrolysis the samples could be retrieved until day 45 for SEC determination. Past this time, it was difficult to collect the samples because they were heavily degraded. Since the abiotic phase or hydrolytic degradation is the rate limiting step for initiating the PLA biodegradation, the hydrolysis experiment was conducted separetely to decouple the abiotic phase and study the degradation behavior [49,70]. The molecular weight decreased during the first 21 days proving that the hydrolytic degradation is the main contributor towards the initial degradation of PLA as previously demonstrated by others [71,72]. The *M*_n_ reduction rate (*k*) for the films was obtained by fitting the experimental data to a simplified first order reaction of the form *M*_n_/*M*_no_ = exp(−*kt*), where *M*_no_ is the initial *M*_n_ and *t* is the time. Irrespective of the testing media, the *M*_n_ reduction of all films was not significantly different as shown in Table 1. These results are in contrast to the findings published in the literature for samples added with nanoclays [68,69,73]. Paul et al., found that the *M*_n_ of PLA reduced by 40% whereas for the PLA nanocomposites reduced by 70% with respect to the initial molecular weight [38]. Ray and Okamoto also reported that the reduction in *M*_n_ of PLA and PLA nanocomposites was the same even though they did not control for the initial molecular weight, thickness or crystallinity of the samples implying that the rate of molecular weight change is independent of the above mentioned factors [41]. This is in accordance with the result we achieved.

The presence of OMMT is reported to enhance the absorption of water in the PLA matrix. The presence of hydrophilic nanoclays improves the sorption of water, in turn improving the hydrophilicity of the resulting matrix. Table 2 presents the rate constants *k* (d^−1^) for PLA, PLA QAC, PLA MMT and PLA OMMT films exposed to different media. As seen, the rates were similar regardless of the media. This can be attributed to the same starting conditions, like *M*_no_, thickness and %*X*_c_. At high molecular weight, there is restricted segmental mobility of the backbone chains and as such less access to the hydroxyl and hydrophilic terminal carboxyl groups for the water to access. However, the presence of nanoclays may also act as an anchor and present a torturous path, thereby hindering the diffusion of water into the PLA OMMT matrix. A competitive balance may be achieved between the higher absorption of water and slow diffusion of water resulting in similar hydrolysis rates for both samples. Though PLA QAC had an advantage of low starting molecular weight as compared to the other films, by the end of the test there was no significant difference in the hydrolysis rates. Overall, although the addition of nanoclays may increase water sorption, it may delay water diffusion into the bulk of PLA resulting in an identical hydrolysis and biodegradation.

We demonstrated that, considering the power of the running experiments (i.e., number of samples and level of detection *p* < 0.05), there were no differences on the initial hydrolytic rate and the final obtained biodegradation of the samples. More studies should be conducted to further decouple the underlying degradation mechanism of PLA and PLA nanocomposites in water and inoculated and non-inoculated vermiculite. This information should be useful to inform the biodegradation of PLA in different environments such as compost, soil and marine environments.

## 4. Conclusions

The effect of MMT, OMMT and surfactant on the hydrolytic degradation and biodegradation of PLA was assessed by producing PLA based films with the same initial parameters (i.e., weight number molecular weight, thickness and crystallinity). The films were evaluated in an inhouse built DMR system to understand the effect of nanoclays and surfactant on the biodegradation of PLA. A hydrolysis experiment was conducted separately to understand the abiotic phase. The results obtained indicated that if the starting parameters are similar, regardless of the presence of QAC, MMT and OMMT no significant difference was observed in the abiotic phase until day 30 for all the films. This result was confirmed by the hydrolysis test. The incorporation of nanoclays did not produce any remarkable effect on PLA film biodegradation, perhaps due to the competitive balance reached between the absorption and diffusion of water in the PLA matrix. Further studies are recommended to fully understand the biodegradation of PLA and its composites in different environments. Studies in compost environment and with equal weight of PLA samples could provide additional insights on the degradation mechanism. Visual tracking of the macro structure of the films during biodegradation using SEM analysis could provide an understanding about the formation of microplastics.

## Figures and Tables

**Table 1:** Initial characterization of polylactic acid (PLA), PLA QAC, PLA montmorillonite (MMT) and PLA Organomodified montmorillonite (OMMT) films.

Films	*M*_no_, kDa	*M*_wo_, kDa	SEM-Thickness (µm)	Crystallinity, *X*_c_ (%)
PLA	109.8 ± 3.1 ^a^	185.5 ± 9.3 ^a^	19.8 ± 0.4 ^a^	3.7 ± 4.7 ^a^
PLA QAC	87.6 ± 4.2 ^b^	128.6 ± 4.8 ^b^	19.0 ± 3.8 ^a^	1.0 ± 1.0 ^a^
PLA MMT	111.1 ± 4.0 ^a^	160.4 ± 5.7 ^a^	17.4 ± 1.0 ^a^	2.3 ± 2.1 ^a^
PLA OMMT	114.3 ± 11.8 ^a^	170.4 ± 5.8 ^a^	21.5 ± 1.3 ^a^	1.7 ± 1.2 ^a^

Values with different letters within columns are statistically different (α = 0.05 Tukey-Kramer Test).

**Table 2:** Rate constant *k* (d^−1^) for PLA, PLA QAC, PLA MMT and PLA OMMT and exposed to different media.

*k* (d^−1^) for Films in Different Media			
Films	Inoculated Vermiculite	Non-inoculated Vermiculite	Water
PLA	0.0735 ± 0.0137 ^a,A^	0.1035 ± 0.0120 ^a,A^	0.0828 ± 0.0081 ^a,A^
PLA QAC	0.0874 ± 0.0149 ^a,A^	0.0996 ± 0.0086 ^a,A^	0.1060 ± 0.0174 ^a,A^
PLA MMT	0.0843 ± 0.0113 ^a,A^	0.0986 ± 0.0085 ^a,A^	0.0973 ± 0.0126 ^a,A^
PLA OMMT	0.1087 ± 0.0211 ^a,A^	0.0959 ± 0.0077 ^a,A^	0.1002 ± 0.0110 ^a,A^

Values with different lowercase letters (a) in a column and values with different uppercase letters (A) in a row are statistically different (α = 0.05 Tukey-Kramer Test).

## Supplementary Materials

The supplementary materials are available online at https://www.mdpi.com/2073-4360/12/2/311/s1.
