# Supplementary material for: Effect of Nano-Clay and Surfactant on the Biodegradation of Poly(Lactic Acid) Films
Source: Polymers (Basel). 2020 Feb 3;12(2):311. doi: 10.3390/polym12020311 (PMC7077407; doi:10.3390/polym12020311)
Supplement: Supplementary file 1 [file polymers-12-00311-s001.pdf]

Supporting information

Effect of nano-clay and surfactant on the biodegradation of poly(lactic acid) films

Pooja C. Mayekar[1], Edgar Castro-Aguirre[1], Rafael Auras[1,*], Susan Selke[1], Ramani Narayan[2]

[1] School of Packaging, Michigan State University, East Lansing, MI, 48824-1223, U.S.
[2] Department of Chemical Engineering and Material Science, East Lansing, MI, 48824-1223, U.S.
* Correspondence: aurasraf@msu.edu; Tel.: +1-517-432-3254

Table of Contents

S1 Production of Masterbatch and Nanocomposite Films ……………………………3

S2 Suggested mechanism for PLA and QAC reaction …………………………….....4

S3 Characterization of the Films ……………………………………………………5

S4 Characterization of PLA OMMT Film …..…………………………………….…...9

S5 Schematic representation of the migration cell and Hydrolysis setup..…………...11

S6 Change in crystallinity for all films in water ………………...12

S7 References...…………………..………………………………………………………14

S1 Production of Masterbatch and Nanocomposite Films

PLA pellets were dried along with OMMT and MMT clay powder at 50°C for 24 h in a vacuum oven (Sheldon Manufacturing, Inc; Cornelius, OR, USA) to remove any moisture present prior to processing. PLA nanocomposite films were produced in a two-tier process. In the first step, masterbatches were produced in a co-rotating Century ZSK 30 twin-screw extruder (Century Extruders, Traverse City, MI, USA) having a L/D ratio of 42:1 to achieve uniform dispersion. PLA was mixed with 5% wt. of MMT, 7% wt. of OMMT (to have approximately the same amount of MMT) and 20% wt. of QAC in a bag. To produce PLA MMT, PLA OMMT and PLA QAC masterbatches the twin-screw extruder was operated at a temperature range of 140/150/160/160/160/170/170/170/160/160 ℃ from the feed throat to the die, with a feed rate of 70 g/min and a residence time of 2 min. The extrudate coming out of the circular die was passed through a water bath for cooling before feeding onto a BT 25 pelletizer (Scheer Bay Co., Bay City, MI, USA). The pelletized masterbatches were then placed in an oven at 50°C for 24 h to remove any residual water present and later stored in a freezer at -21°C until further use. The second step involved producing films using a RandCastle RCP-0625 Multi-Layer Cast film extruder (Randcastle Extrusion Systems, Inc., Cedar Grove, NJ, USA) with L/D ratio of 24:1, volume of 34 cm^3 and 15.875 mm single screw. Table S1 provides the extrusion conditions used to produce the PLA, PLA OMMT, PLA MMT and PLA QAC films. After production, the films were stored at a temperature of -15°C before further characterization.

Table S1. Processing conditions for the cast film extrusion

	Extrusion Conditions			
Materials	Temperature Profile (°C) (Zone 1 to die)	Screw speed (rpm)	Nip roller (rpm)	Winding roller (rpm)
PLA	193/229/229/229/229/204/204	35	40	15
PLA OMMT	139/150/160/160/160/170/160	30	30	12
PLA MMT	139/150/160/160/160/170/160	30	40	12
PLA QAC	132/143/152/152/152/163/160	30	40	12

S2 Suggested mechanism for PLA and QAC reaction

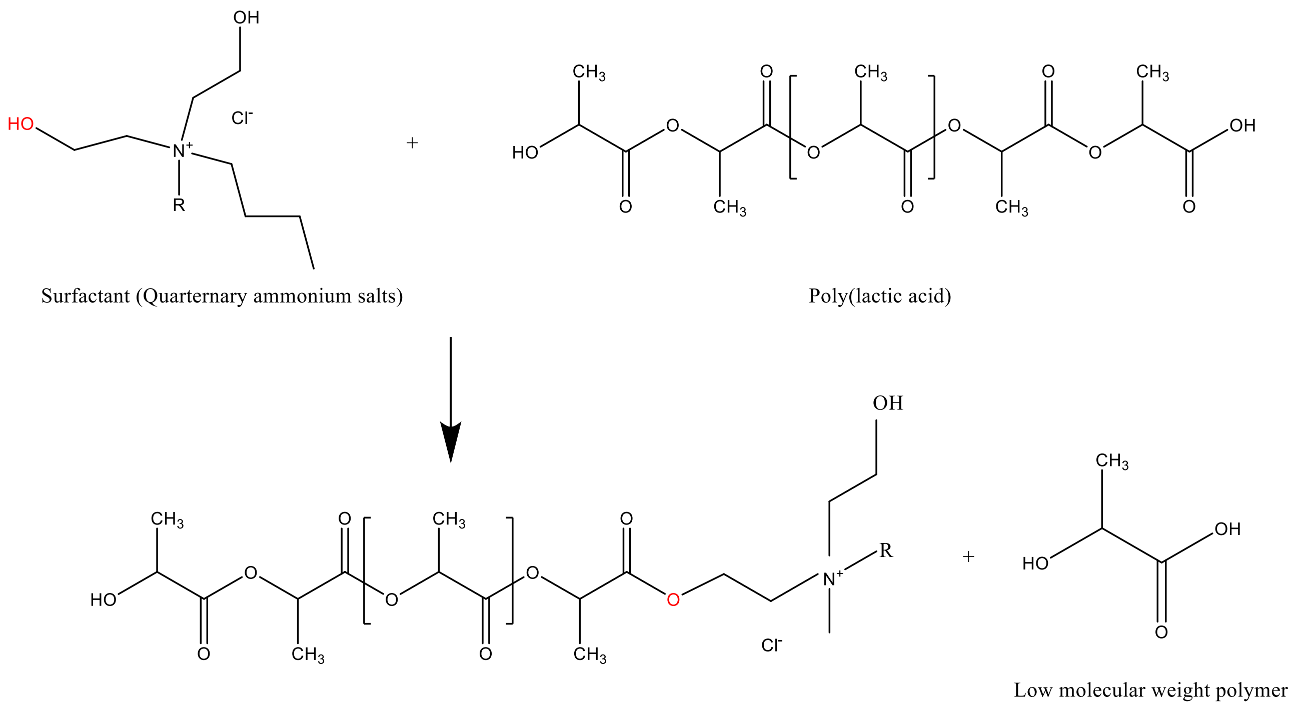

Scheme 1. Suggested mechanism of reaction between PLA and surfactant.

S3 Characterization of the Films

CHN Elemental Analysis: A PerkinElmer 2400 Series II CHNS/O Elemental Analyzer (PerkinElmer Inc., Shelton, CT, USA) was used to determine the carbon, hydrogen, and nitrogen content of the different test materials as shown in Table S2.

Table S2. Amounts of carbon, hydrogen, and nitrogen content determined by CHNS/O Elemental Analyzer (%)

Materials	Carbon, wt %	Hydrogen, wt %	Nitrogen, wt %
QAC	17.0 ± 0.0	2.2 ± 0.1	0.9 ± 0.0
OMMT	17.2 ± 0.0	3.5 ± 0.0	0.9 ± 0.0
MMT	0.2 ± 0.0	1.3 ± 0.1	0.0 ± 0.0
PLA	51.2 ± 0.1	5.6 ± 0.0	0.0 ± 0.0
PLA QAC	50.3 ± 0.3	5.6 ± 0.0	0.1 ± 0.0
PLA OMMT	49.1 ± 0.0	5.5 ± 0.0	0.1 ± 0.0
PLA MMT	48.5 ± 0.1	5.5 ± 0.0	0.0 ± 0.0

Thermogravimetric Analysis (TGA): A Q-50 thermogravimetric analyzer (TA Instruments Inc., New Castle, DE, USA) was used to determine the degradation temperature of the PLA, PLA QAC, PLA MMT and PLA OMMT films. The film samples were tested to a temperature of 600°C at a ramp rate of 10°C/min under pure nitrogen. Figure S1 depicts the thermograms for PLA, PLA QAC, PLA MMT and PLA OMMT films.

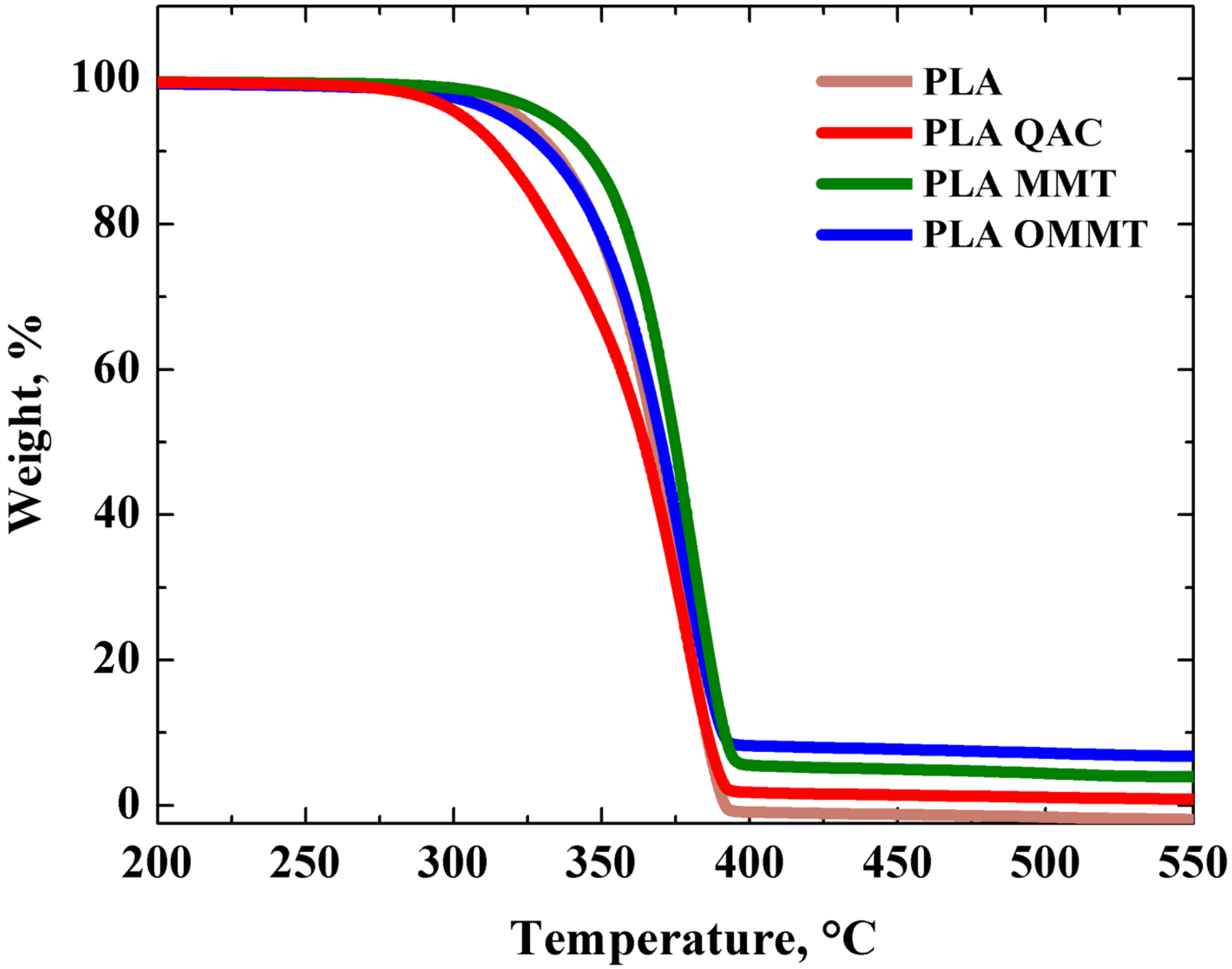

Figure S1. TGA of PLA, PLA QAC, PLA MMT and PLA OMMT films.

Direct Scanning Calorimetry (DSC): A Q100 differential scanning calorimeter (TA Instruments Inc., New Castle, DE, USA) was used to determine the glass transition temperature (T_g), melting temperature (T_m), crystallization temperature (Tc), enthalpy of cold crystallization process (ΔH_c), and total enthalpy of melting peaks (ΔH_m). The samples were heated to 180°C at a rate of 10°C/min. The thermograph obtained was analyzed using the software Thermal Universal Analysis 2000, V4.5 (TA Instruments Inc., New Castle, DE, USA). Figure S2 shows an example of the DSC curve for all the films while Table S3 shows the thermal properties of the PLA, PLA QAC, PLA MMT and PLA OMMT films.

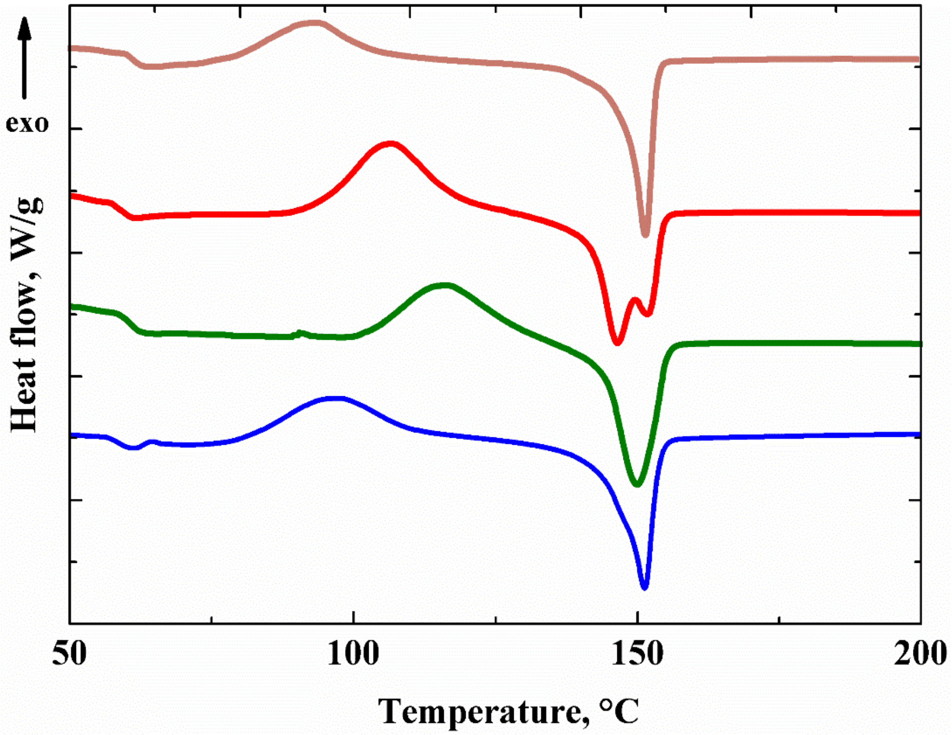

Figure S2. DSC (1st cycle) (b) of PLA, PLA QAC, PLA MMT and PLA OMMT films.

Table S3. Thermal properties of PLA, PLA QAC, PLA MMT and PLA OMMT films

Materials	T_g, °C	T_m, °C	T_c, °C	χ_c, %
PLA	60.1±0.5[a]	149.9±1.6[a]	93.6±0.5[a]	1.6±1.5[a]
PLA QAC	58.3±1.5[a]	149.6±2.7[a]	102.2±3.9[b]	1.1±1.0[a]
PLA OMMT	59.7±0.5[a]	151.1±0.6[a]	96.8±3.2[a]	3.5±2.1[a]
PLA MMT	58.5±0.5[a]	148.2±1.3[a]	112.4±3.6[c]	1.7±1.2[a]

Note: Values with different letters within columns are statistically different (α = 0.05, Tukey-Kramer Test.)

Film Thickness (*t*): The thickness of the films was determined using a digital micrometer model N# 49-70-01-0001 (Testing Machines Inc, New Castle, DE, USA). Since the nanoclays form a bump like structure, the thickness measured by the digital micrometer for PLA MMT and PLA OMMT was unreliable and scanning electron microscopy (SEM) was also used to measure the thickness of PLA MMT, PLA OMMT films. Samples were mounted on aluminum stubs using adhesive tapes and the morphology was analyzed under a SEM JOEL 6610LV (JEOL, Tokyo,

Japan) microscope. Table S4 provides the comparison of thickness of the PLA, PLA QAC, PLA MMT and PLA OMMT films as measured by digital micrometer and SEM. Figure S3 shows examples of the SEM micrographs.

Table S4. Micrometer and SEM thickness t for PLA, PLA QAC, PLA MMT and PLA OMMT films

Materials	Micrometer - Thickness (μm)	SEM - Thickness (μm)
PLA	19.30 ± 1.27^{a}	19.78 ± 0.39^{a}
PLA QAC	19.03 ± 1.69^{a}	18.97 ± 3.81^{a}
PLA OMMT	71.12 ± 6.35^{b}	21.47 ± 1.30^{a}
PLA MMT	77.72 ± 8.16^{b}	17.39 ± 1.01^{a}

Values with different letter within a column are statistically different (α = 0.05 Tukey-Kramer Test)

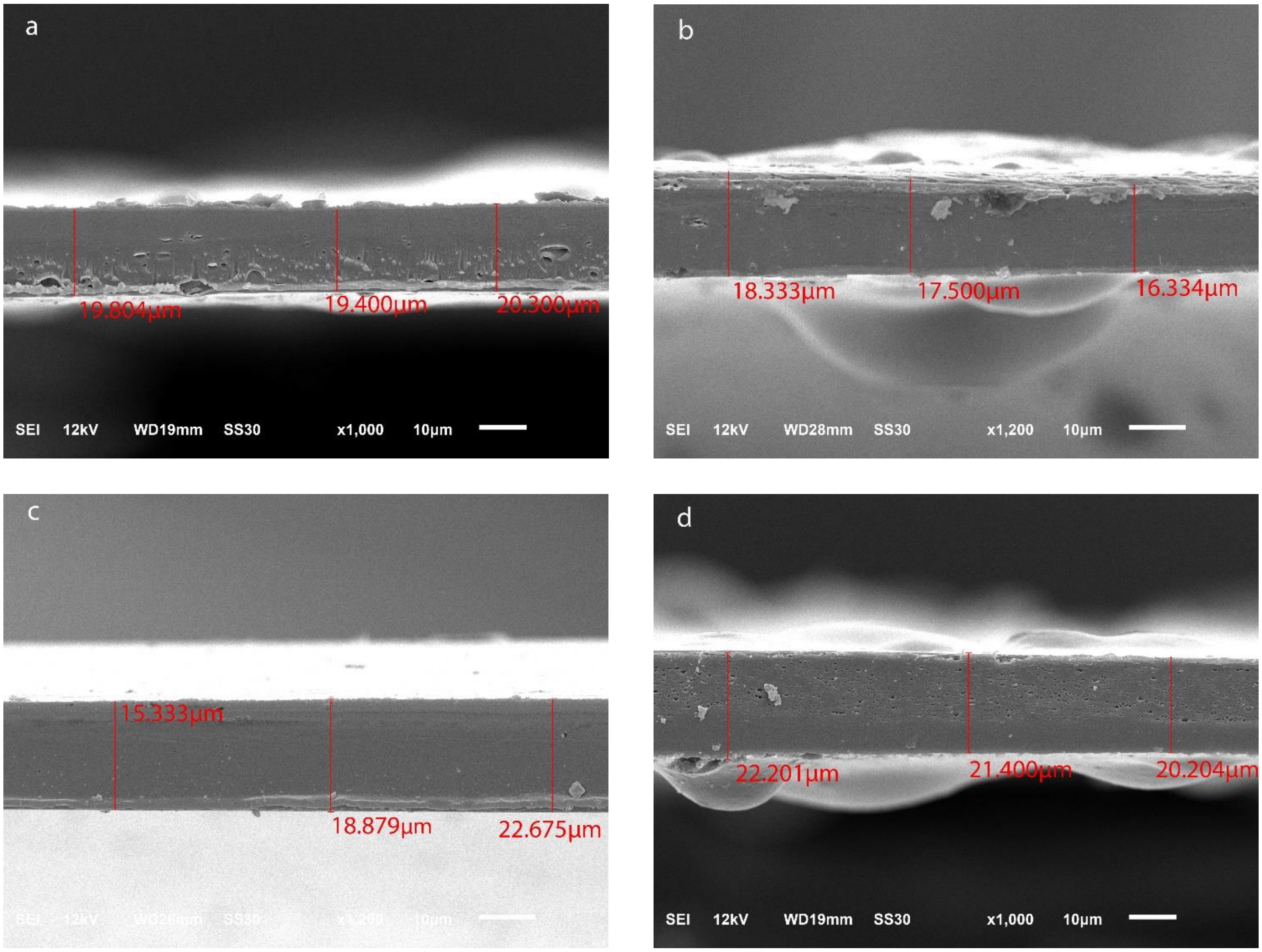

Figure S3. SEM micrographs of a) PLA, b) PLA MMT, c) PLA QAC and d) PLA OMMT films.

S4 Characterization of PLA OMMT Film

Fig. S4 shows the XRD patterns of OMMT clay powder, MMT clay powder, PLA, PLA MMT, and PLA OMMT films. This method is used to determine the presence and dispersion of MMT and OMMT nanoclays in the PLA matrix. The resulting nanocomposite films can be either intercalated or exfoliated, depending on the nature of dispersion of the nanoclays. Intercalated morphologies are formed when the polymer chains infiltrate the silicate layers of the clay, while exfoliation occurs when the silicate layers are completely delaminated and dispersed randomly in the polymer matrix [1]. The formation of intercalated morphologies is demonstrated by shifting of the peak to a smaller angle and increase in the interlayer spacing while exfoliated is indicated by the absence of the peak. For the OMMT clay, the diffraction peak was at $2\theta = 4.76°$ corresponding to *d*-spacing 18.53 Å , while for MMT clay the peak was observed at $2\theta = 7.2°$ corresponding to *d*-spacing 11.23 Å . The *d*-spacing of OMMT increased to 18.53 Å indicating that the surface of MMT was modified by means of ion exchange of the surfactant [2]. As seen in Fig. S4, in case of PLA OMMT film, the formed nanocomposite is intercalated in nature, which is represented by the shift of peak from $2\theta = 4.76°$ (*d*-spacing 18.53 Å) to $2\theta = 2.44°$ (*d*-spacing 36.16 Å) *i.e.*, to the left of the pristine clay. It is difficult to achieve full exfoliation in a laboratory setting as well as in industrial settings and for most part the nanocomposites are a mixture of intercalated and exfoliated morphologies, which is also referred to as a disordered morphology or orderly exfoliated morphology [3].

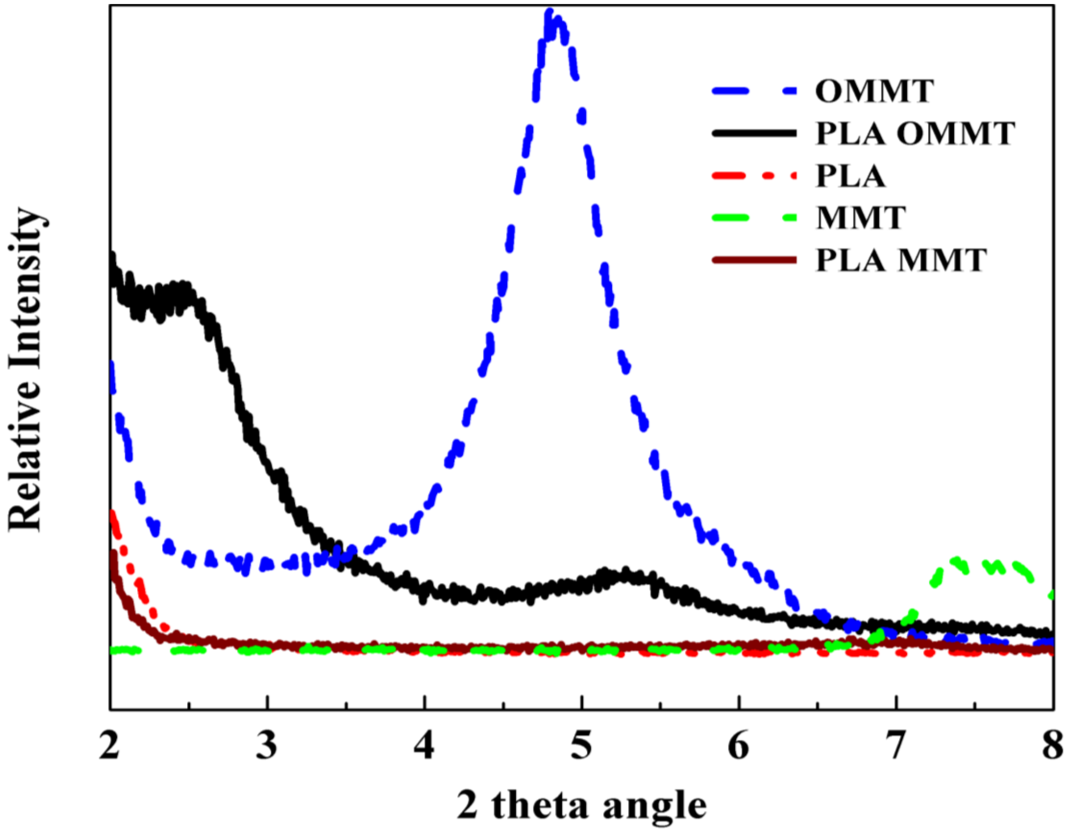

Figure S4. XRD spectra for OMMT and MMT clay powder ,PLA OMMT, PLA MMT and PLA films.

S5 Schematic representation of the migration cell and hydrolysis test

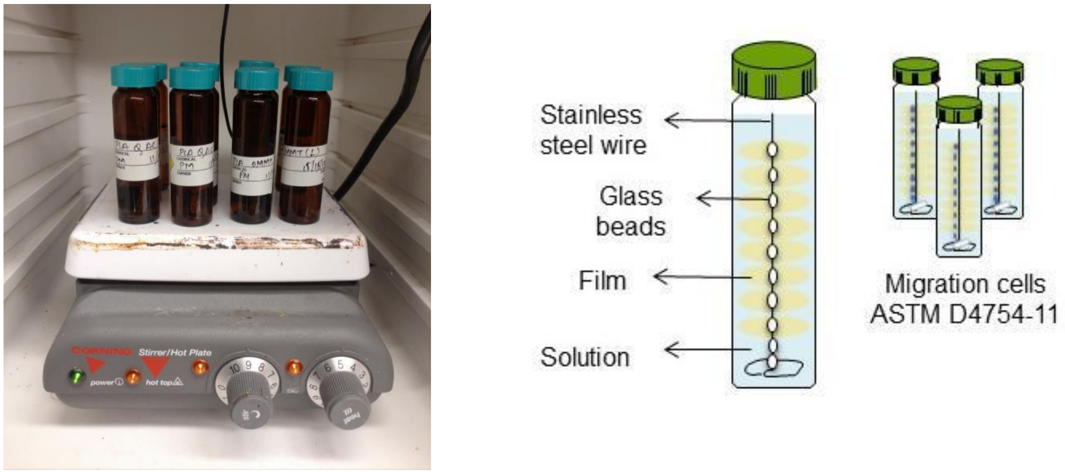

Figure S5. Hydrolysis setup in the lab and migration cell.

S6 Change in crystallinity for all films in water

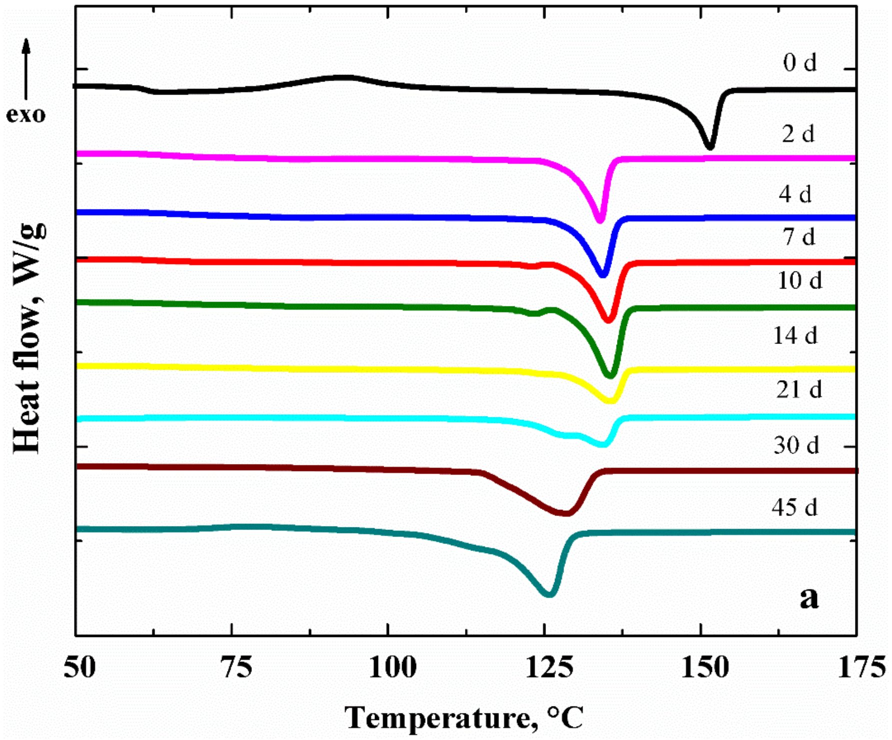

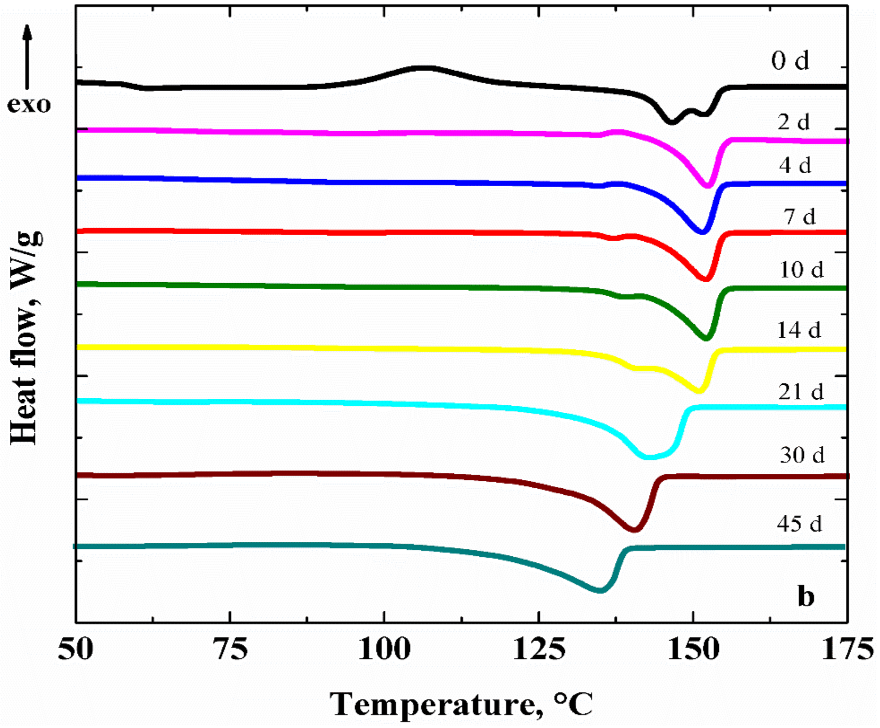

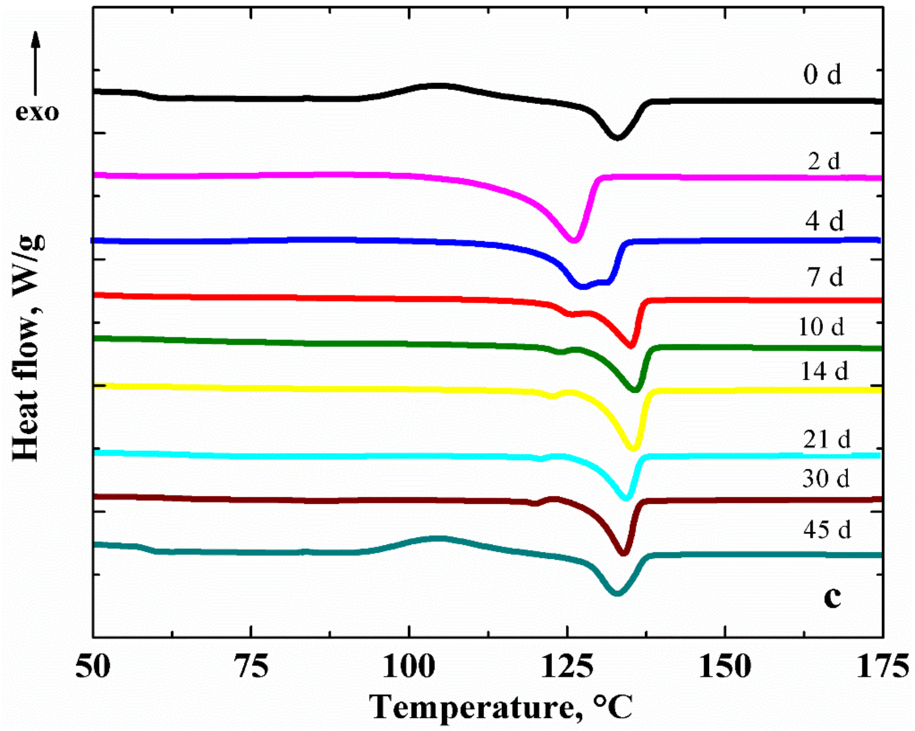

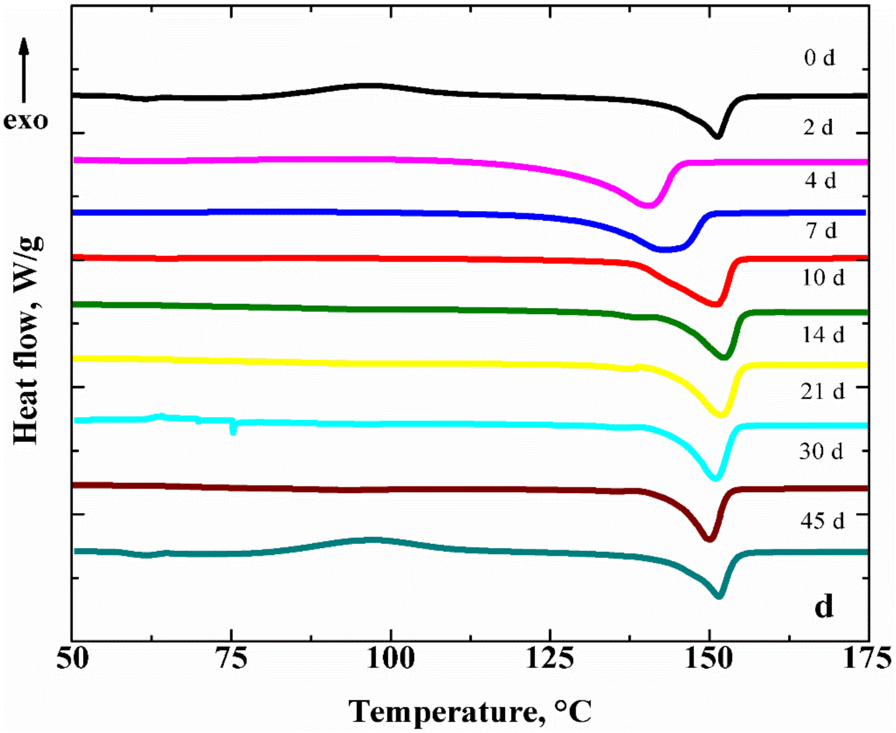

Figure S6. DSC thermograms of a) PLA b) PLA QAC c) PLA MMT and d) PLA OMMT in water at 60° C (1st cycle thermal scan). The numbers on the thermogram indicate the day immersed.

S7 References

[1] H.M.C. de Azeredo, Nanocomposites for food packaging applications, *Food Res. Int.* **2009**, *42*, 1240–1253. https://doi.org/10.1016/j.foodres.2009.03.019.

[2] A. P. Kumar, D. Depan, N. Singh Tomer, and R. P. Singh, "Nanoscale particles for polymer degradation and stabilization—Trends and future perspectives," *Prog. Polym. Sci.*, **2009**, *34*, 479–515.

[3] J.-M. Raquez, Y. Habibi, M. Murariu, and P. Dubois, "Polylactide (PLA)-based nanocomposites," *Prog. Polym. Sci.*, **2013**, *38*, 1504–1542.
